# Delineation of a minimal topoisomerase II binding protein 1 for regulated activation of ATR at DNA double-strand breaks

**DOI:** 10.1016/j.jbc.2022.101992

**Published:** 2022-04-28

**Authors:** Kenna Ruis, Oanh Huynh, Katrina Montales, Nina A. Barr, W. Matthew Michael

**Affiliations:** Department of Biological Sciences, Molecular and Computational Biology Section, University of Southern California, Los Angeles, California, USA

**Keywords:** cell biology, checkpoint control, DNA damage, protein kinase, DNA repair, TOPBP1, ATR, CHK1, BRCT, DNA double-strand break, AAD, ATR activation domain, ATR, ataxia telangiectasia and Rad3-related, BRCT, BRCA1 C-terminal repeat, DMAX, DSB-mediated ATR activation in Xenopus, DSBs, DNA double-strand breaks, HSS, high-speed supernatant, IVTT, *in vitro* transcription and translation, P-CHK1, phosphorylated CHK1, TOPBP1, Topoisomerase II binding protein 1, UAS, upstream activator sequence, XEE, Xenopus egg extract

## Abstract

Topoisomerase II Binding Protein 1 (TOPBP1) is an important activator of the DNA damage response kinase Ataxia Telangiectasia and Rad3-related (ATR), although the mechanism by which this activation occurs is not yet known. TOPBP1 contains nine copies of the BRCA1 C-terminal repeat (BRCT) motif, which allows protein–protein and protein–DNA interactions. TOPBP1 also contains an ATR activation domain (AAD), which physically interacts with ATR and its partner ATR-interacting protein (ATRIP) in a manner that stimulates ATR kinase activity. It is unclear which of TOPBP1’s nine BRCT domains participate in the reaction, as well as the individual roles played by these relevant BRCT domains. To address this knowledge gap, here, we delineated a minimal TOPBP1 that can activate ATR at DNA double-strand breaks in a regulated manner. We named this minimal TOPBP1 “Junior” and we show that Junior is composed of just three regions: BRCT0-2, the AAD, and BRCT7&8. We further defined the individual functions of these three regions by showing that BRCT0-2 is required for recruitment to DNA double-strand breaks and is dispensable thereafter, and that BRCT7&8 is dispensable for recruitment but essential to allow the AAD to multimerize and activate ATR. The delineation of TOPBP1 Junior creates a leaner, simplified, and better understood TOPBP1 and provides insight into the mechanism of ATR activation.

DNA double-strand breaks (DSBs) are a potentially lethal form of DNA damage that must be repaired in an accurate manner to maintain genome stability. DSBs activate a variety of protein kinases that go on to organize repair and to regulate cell cycle progression (reviewed by (1, 2, 3, 4)). Among these kinases, ataxia-telangiectasia mutated and DNA-dependent protein kinase have been heavily studied and the mechanism for their activation by DSBs is understood (2, 3). By contrast, very little is known about how another important kinase, Ataxia Telangiectasia and Rad3-related (ATR), is activated by DSBs. ATR is crucial for the cell’s ability to slow the cell cycle upon DSB induction, as it phosphorylates and activates the CHK1 kinase, which goes on to delay entry into mitosis *via* regulation of CDC25 (1). ATR has also been implicated in promoting DNA end resection (5), a critical step in the error-free repair of DSBs. ATR is thus crucial for the cell’s ability to survive DSBs, which underscores the need to unravel the mechanism.

Vertebrate ATR is activated at DSBs by the TOPBP1 protein (6, 7, 8). Structurally, TOPBP1 contains 9 BRCT domains and an ATR activation domain (AAD). BRCA1 C-terminal repeat (BRCT) domains mediate protein–protein interactions, and they also possess DNA-binding activity (reviewed in (9, 10, 11). The classic model for how vertebrate TOPBP1 activates ATR is derived from the studies of stalled replication forks and states that the RAD9-RAD1-HUS1 (9-1-1) clamp is loaded onto 5′-ssDNA/dsDNA junctions by the RAD17-RFC clamp loader. TOPBP1 is then recruited to the stalled fork *via* interaction between the RAD9 “tail” domain and TOPBP1’s BRCT0-2 region. ATR and ATRIP arrive separately, *via* binding of ATRIP to RPA-coated ssDNA, and TOPBP1 activates ATR (reviewed in (1, 2, 3)). While this model is very popular, and is presented in many reviews, the issue of how TOPBP1 is recruited to stalled forks is still not settled as published data show that the interaction with RAD9 is dispensable for TOPBP1 recruitment (12, 13), dispensable for ATR to phosphorylate some of its substrates (14), and that TOPBP1 actually recruits RAD9 and the 9-1-1 complex to stalled forks (15, 16, 17). Adding additional complexity to the mechanism for TOPBP1-mediated activation of ATR are recent findings showing that human TOPBP1 undergoes liquid-liquid phase separation to create large, micron-sized condensates that, in an unknown manner, amplify ATR signaling (18).

In addition to promoting ATR signaling, TOPBP1 plays numerous other roles in the cell (reviewed in (19)). We have previously shown that it is essential for the initiation of DNA replication (20), and others have shown that it regulates transcription and that it plays ATR-independent roles in DNA repair (reviewed in (19, 21)). The multiple functions, and essential nature, of TOPBP1 make it a difficult protein to study using cultured human cells as the model system. Depletion of TOPBP1 kills proliferating cells and, prior to death, the cells enter a pathological state. In addition, loss of TOPBP1 causes pleiotropic effects that render analysis of any one particular function difficult. To get around these issues, we recently developed an experimental tool called DMAX for DSB-mediated ATR activation in *Xenopus* egg extracts (8). One of the great strengths of the DMAX system is that the essential nature of TOPBP1 is no longer a factor; *Xenopus* egg extract (XEE) does not enter a pathological state upon loss of TOPBP1. Furthermore, the issues of pleiotropic effects are also largely removed as there is no DNA replication or transcription happening in our DMAX system. Lastly, we have shown that TOPBP1 is solely responsible for activating ATR at DSBs in the DMAX system, and thus issues with redundant activators, such as the ETAA1 protein (22), do not exist. DMAX is thus streamlined for the analysis of the role of TOPBP1 in ATR signaling at DSBs. In this work, we used DMAX to delineate a minimal TOPBP1, termed Junior, that is still competent to activate ATR in a regulated manner, that is, specifically at DSBs. Junior is composed of just three regions of TOPBP1, BRCT0-2, the AAD, and BRCT7&8, and we go on to identify specific functions for each of these three regions.

## Results

### The BRCT0-2 region plays the central role in recruitment of TOPBP1 to DSBs *via* an indirect mode of DNA binding.

**Figure 1:**
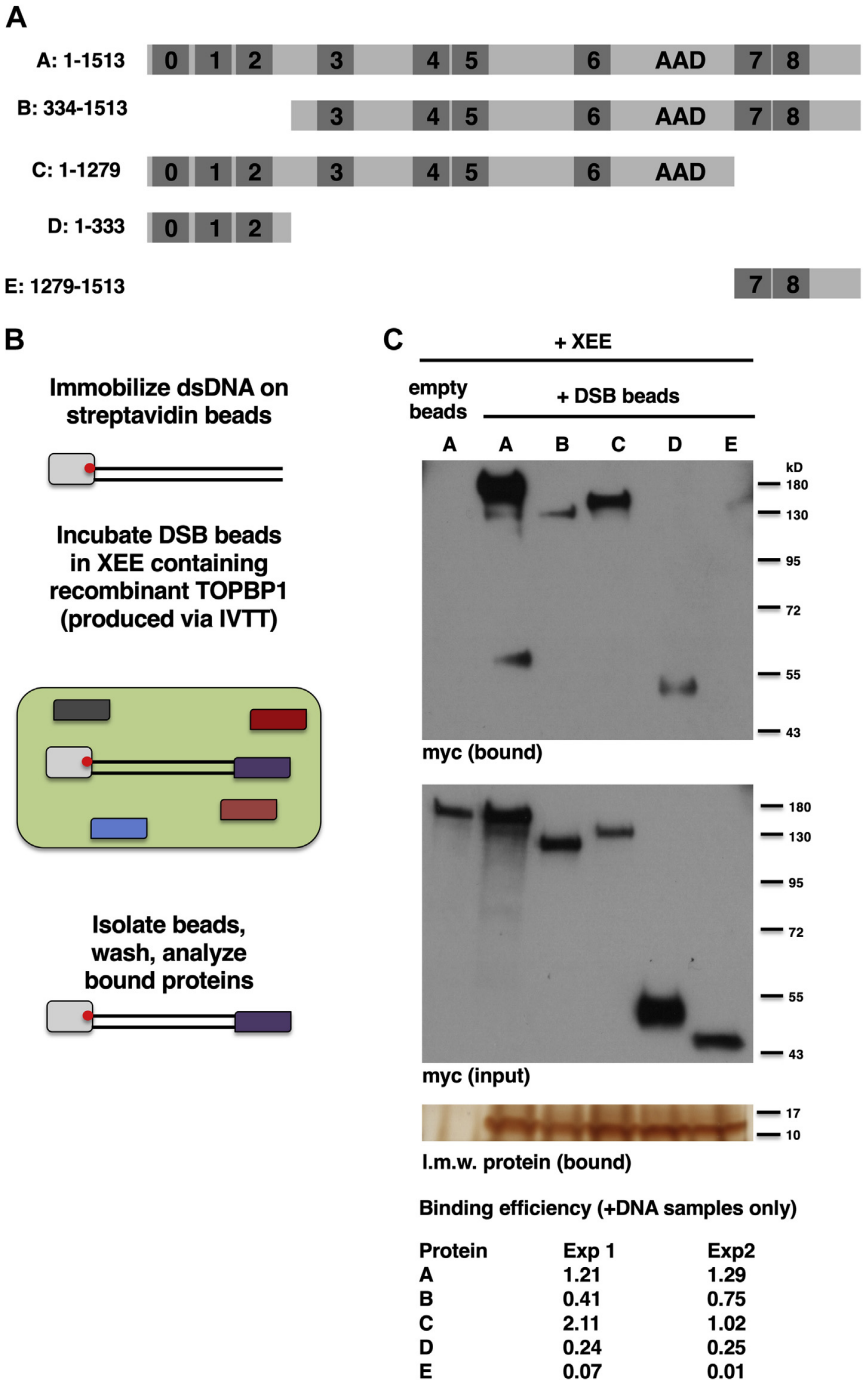
**Deletion analysis of TOPBP1 recruitment to DSBs.***A*, schematic summarizing the TOPBP1 proteins that were tested for binding to DSBs. The proteins are referred to by the letters A-E, at *left*, and also shown are the amino acid residues corresponding to each. *B*, schematic summarizing the DSB-binding assay. *C*, a representative experiment testing the ability of TOPBP1 deletion mutants to bind DSBs in XEE is shown. IVTT-expressed and myc-tagged proteins were mixed with XEE at 1 part IVTT lysate (5 μl) to 4 parts XEE (20 μl). DSB beads (600 fm of 5kb dsDNA in a volume of 5 μl) were then added and the samples were incubated at room temperature for 30 min. The beads were then isolated back out of the extract, washed, and probed for occupancy of the indicated TOPBP1 deletion mutant. Panel “myc bound” refers to material that was bound to the DSB beads and the signal represents 20% of the total bound material. Panel “myc input” refers to a sample of the total extract taken prior to addition of the DSB beads, and the signal represents 0.5% of the total amount present in the reaction. Panel “low molecular weight protein bound” is a silver-stained gel showing low molecular weight protein, likely histone, that bind DNA beads and not empty beads. This is used as a control for equal isolation of the DSB beads across the sample set. The experiment shown is representative of two independently performed replicates. DSBs, DNA double-strand breaks; IVTT, *in vitro* transcription and translation; TOPBP1, Topoisomerase II binding protein 1; XEE, Xenopus egg extract.

For ATR activation at DSBs, TOPBP1 contains three basic properties. One, it is recruited to DSBs. Two, once at the DSB, it interacts with ATR-ATRIP to stimulate ATR. Three, TOPBP1 is regulated so that it only activates ATR at DSBs, and not constitutively. The goal of this study was to define the sequence elements that allow for these basic properties. We began by examining how TOPBP1 is recruited to DSBs. Recent work from our group had used BRCT “misfolding” mutants to map which BRCT domains participate in TOPBP1’s activation of ATR (8, 23). Such mutants change a highly conserved hydrophobic residue, one that is buried in the interior of the folded BRCT domain, to a charged residue (arginine). As detailed in (8, 23), these misfolding mutants inactivate the BRCT domain containing them. For recruitment to DSBs, we found that misfolding mutants in BRCT domains 1,2,7, and 8 attenuated recruitment (8). To explore this further, we generated a series of deletion mutants designed to test the requirements for the BRCT0-2 and 7&8 regions in binding of TOPBP1 to DSBs (Fig. 1*A*). To do so, we used a previously described DSB-binding assay (8), see Fig. 1*B*). In brief, 5 kb PCR fragments containing a biotin moiety on one end are immobilized on streptavidin beads. Proteins of interest are then produced *via in vitro* transcription and translation (IVTT) in rabbit reticulocyte lysates and added to XEE at a ratio of 1 part IVTT lysates to 4 parts XEE. DNA beads then join the mix and, after incubation, beads are isolated, washed, and probed by Western blot for the protein of interest. To control for equal isolation of beads, we used silver staining to visualize the binding of low molecular weight proteins, likely histones, to the DSB beads. As shown in Figure 1*C*, WT TOPBP1 could bind DSB beads but not empty beads, as expected. To gain an indication of how efficient the binding of TOPBP1 is to DSB beads, we quantified the signal intensities for both the bound and input bands and expressed these values as the ratio of bound over input (termed “binding efficiency” in Fig. 1*C* and hereafter, see Experimental procedures). A fragment lacking the BRCT0-2 region (mutant B in Fig. 1*A*) showed reduced binding to DSBs, relative to WT, across multiple experiments, while a fragment lacking BRCT7&8 (mutant C) could bind DSBs with a slightly better efficiency, relative to WT (Fig. 1*C*). A fragment corresponding to just the BRCT0-2 region (mutant D) could also bind DSBs, whereas the E mutant, corresponding to just the BRCT7&8 region, did not bind (Fig. 1*C*). Thus, loss of BRCT0-2 renders TOPBP1 less able to bind DSBs, and BRCT0-2 itself binds to DSBs, whereas loss of BRCT7&8 modestly increases TOPBP1’s ability to bind DSBs and cannot itself efficiently bind DSBs. These data highlight the central role that BRCT0-2 plays in the recruitment of TOPBP1 to DSBs and suggest that the contribution of BRCT7&8 is modest at best.

**Figure 2:**
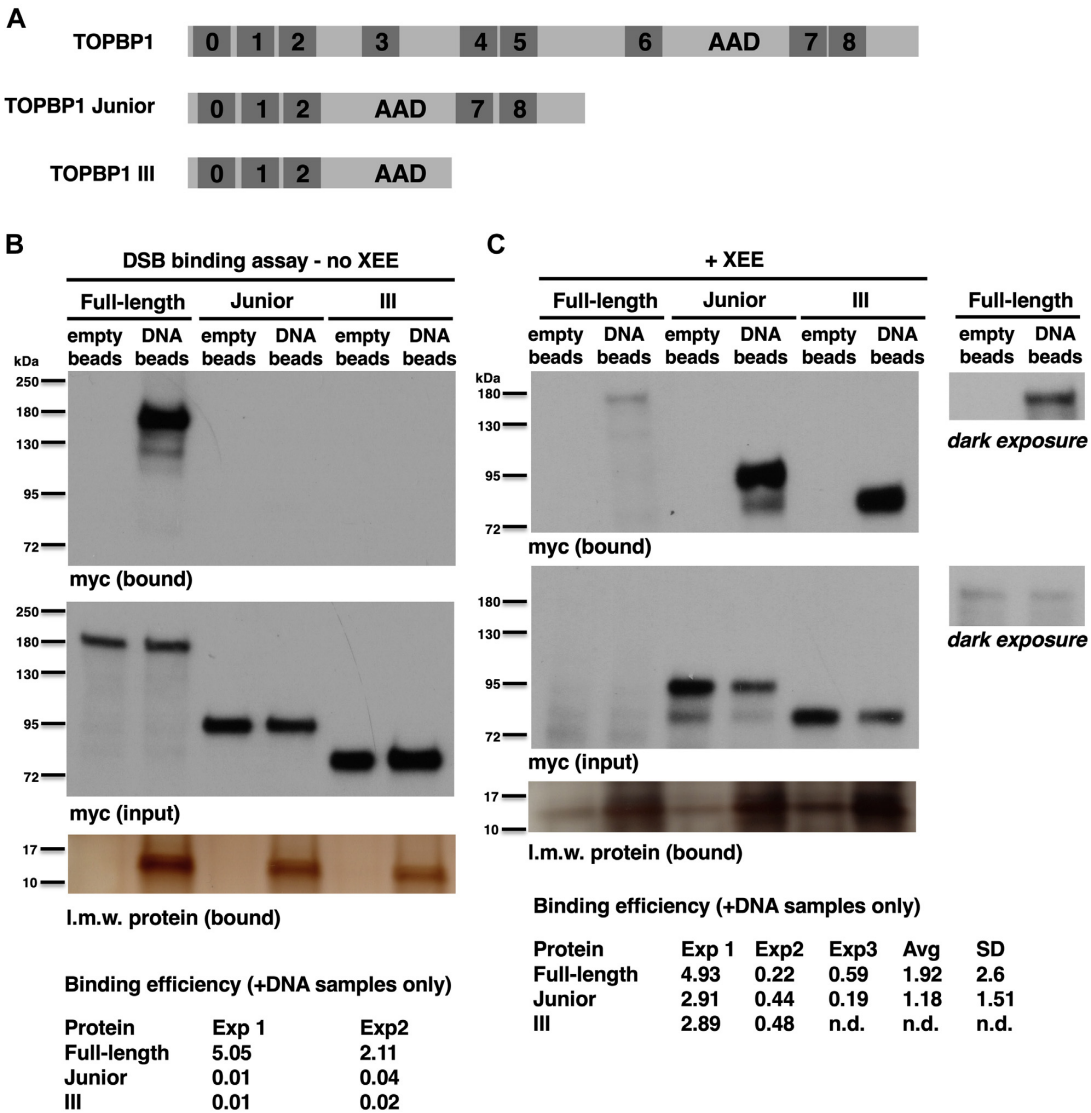
**Smaller, synthetic forms of TOPBP1 require the presence of XEE to bind DSBs.***A*, schematic showing the two synthetic forms of TOPBP1 that were used for DNA- and DSB-binding analysis. *B*, representative experiment testing the ability of the indicated TOPBP1 derivatives to bind dsDNA is shown. There is no XEE in this experiment. Twenty microliters of IVTT lysate expressing the indicated protein was incubated with 600 fmols of 5kb dsDNA immobilized on streptavidin beads (5 μl volume of beads). After 30 min, the beads were isolated, washed, and probed for occupancy of the target protein by virtue of the myc epitope tag. “Empty beads” refers to streptavidin beads lacking DNA. Panel “myc bound” refers to material that was bound to the DSB beads, and the signal represents 20% of the bound material. Panel “myc input” refers to a sample of the total lysate taken prior to addition of the DSB beads, and the signal represents 0.5% of the total amount present in the reaction. The experiment shown is representative of two independently performed replicates. *C*, a representative experiment testing the ability of the indicated TOPBP1 derivatives to bind DSBs in the presence of XEE is shown. This was performed exactly as described in Figure 1*C*. Because the expression of full-length TOPBP1 was weaker than either Junior or III, we included a set of panels showing a darker exposure of the blot, so that the signals for full-length TOPBP1 are easier to see. The experiment shown is representative of three independently performed replicates comparing the binding of full-length TOPBP1 to Junior and two replicates comparing full-length TOPBP1 to Junior and III. AVG refers to average and SD refers to standard deviation. DSBs, DNA double-strand breaks; IVTT, *in vitro* transcription and translation; TOPBP1, Topoisomerase II binding protein 1; XEE, Xenopus egg extract.

Previous work has shown that TOPBP1 can bind DNA directly (24, 25, 26), and it also binds DNA indirectly through interaction with DNA-bound proteins. We, therefore, wanted to determine which mode of binding is relevant to how the BRCT0-2 region interacts with DSBs. To do so, we analyzed DNA binding by full-length, WT TOPBP1 and two synthetic forms of TOPBP1, one which contained the BRCT0-2 region fused to the AAD and BRCT7&8 region (TOPBP1 Junior, Fig. 2*A*) and the other containing the BRCT0-2 region fused to just the AAD (TOPBP1 III, Fig. 2*A*). We then tested the ability of these three proteins to bind dsDNA. For this, proteins were produced *via* IVTT and then DNA beads (the same that were used in Fig. 1*C*) were added directly to the IVTT lysates. After incubation, beads are isolated, washed, and probed for bound proteins by Western blot. Thus, in this assay, there is no XEE involved. As shown in Figure 2*B*, only full-length TOPBP1 could efficiently bind to the DNA. We next performed a DSB-binding assay, as in Figure 1*C*, and thus XEE is now present in the binding reaction. As shown in Figure 2*C*, in this case, all three forms of TOPBP1 could associate with the DSB. We note that the expression of full-length TOPBP1 in the IVTT reactions differed between the experiments shown in Figure 2, *B* and *C*, and in our experience using IVTT to produce full-length TOPBP1, it is not uncommon to see variable expression like this. Based on these data, we conclude that while full-length TOPBP1 can bind DNA directly, the BRCT0-2 region only binds *via* the indirect mode. We base this on the observations that both Junior and III, which contain BRCT0-2, require XEE, and thus additional factors, to bind DSBs whereas the full-length protein does not.

### A minimal TOPBP1 for regulated activation of ATR at DSBs

**Figure 3:**
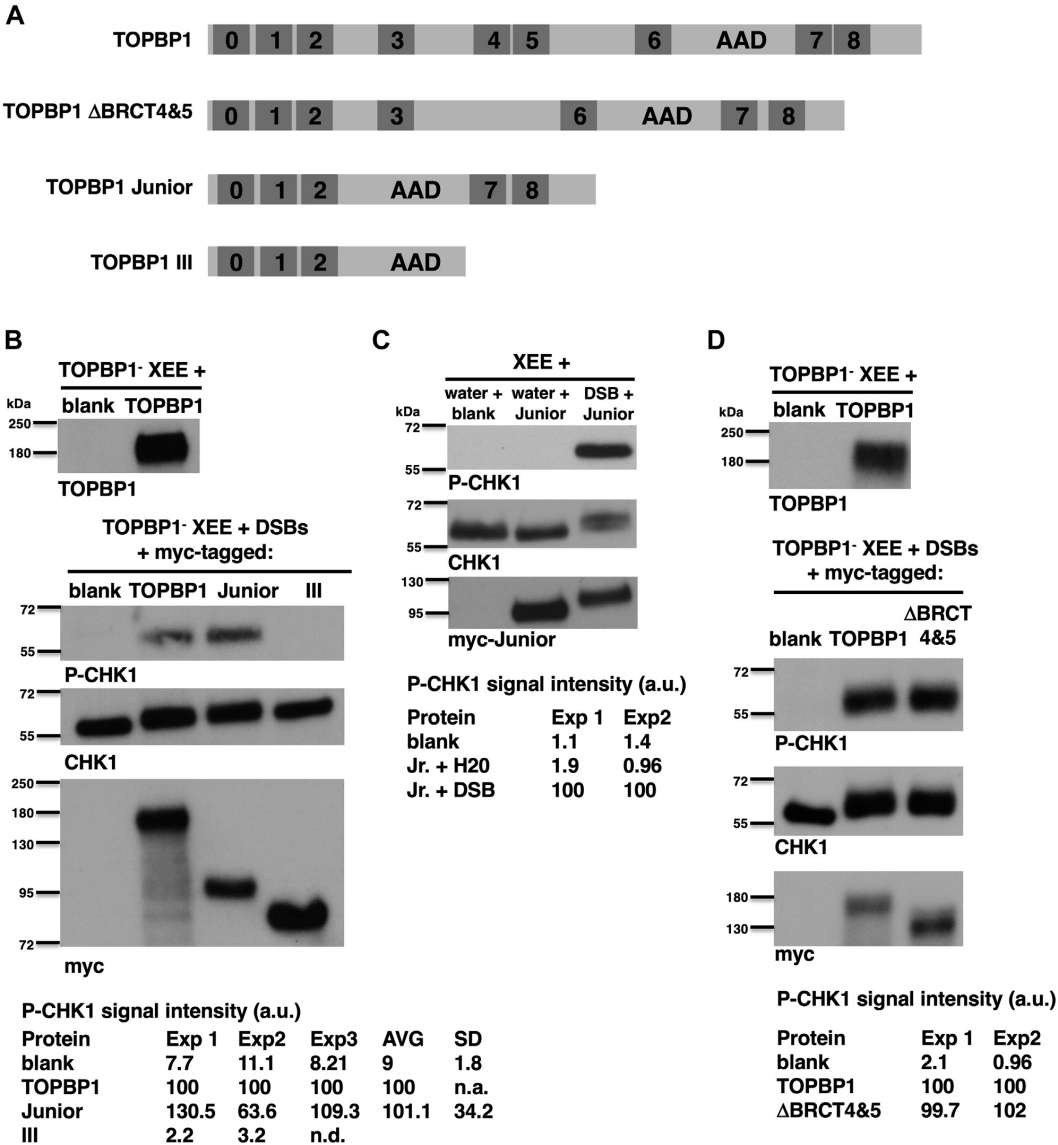
**A minimal TOPBP1 for regulated activation of ATR.***A*, schematic showing the synthetic forms of TOPBP1 that were used for DMAX assays. *B*, a representative experiment testing the ability of the indicated TOPBP1 derivatives to activate ATR. XEE was depleted of endogenous TOPBP1 and supplemented with either unprogrammed IVTT lysate (blank), or IVTT lysates programmed to produce myc-tagged forms of full-length TOPBP1, TOPBP1 Junior, or TOPBP1 III. For each sample, 20 μl of TOPBP1-depleted XEE was combined with 2.5 μl of IVTT lysate. The panel on the *left* shows the blank and full-length TOPBP1 samples probed with an antibody recognizing TOPBP1; this demonstrates that all detectable endogenous TOPBP1 was removed from the XEE. The panels on the *right* show the results of the DMAX assay. All samples received “lambda DSBs”, which is phage lambda DNA digested with the EcoRI restriction enzyme and added at a concentration of 20 ng/μl (see Experimental procedures). After a 30-min incubation, samples were recovered and probed by Western blotting for the indicated proteins. Shown below the blot is quantification of the P-CHK1 signal across multiple replicates. AVG stands for average and SD refers to standard deviation. The experiment shown is representative of three independently performed replicates comparing full-length TOPBP1 to Junior and two replicates comparing full-length TOPBP1 to Junior and III. *C*, a representative experiment testing the requirement for DSBs in TOPBP1 Junior-mediated activation of ATR. XEE (not depleted, 20 μl) was combined with 4 μl of the indicated IVTT lysate and then lambda DSBs were optionally added at 20 ng/μl. After a 30-min incubation, samples were recovered and probed by Western blotting for the indicated proteins. The experiment shown is representative of two independently performed replicates. *D*, a representative experiment examining the ability of the ΔBRCT4&5 mutant to activate ATR is shown. The experiment was performed exactly as described for Part B, above. The experiment shown is representative of two independently performed replicates. ATR, ataxia telangiectasia and Rad3-related; DSBs, DNA double-strand breaks; DMAX, DSB-mediated ATR activation in Xenopus; IVTT, *in vitro* transcription and translation; TOPBP1, Topoisomerase II binding protein 1; XEE, Xenopus egg extract.

Having observed the recruitment of Junior and III to DSBs in XEE, we next asked if either of these synthetic forms could activate ATR on the DSBs. For this, endogenous TOPBP1 was removed from XEE, *via* immunodepletion. We then performed an DMAX assay, where EcoRI-digested lambda DNA is added to XEE as a source of DSBs, and ATR activation is monitored *via* probing for the serine 345-phosphorylated form of the key ATR substrate, CHK1 (P-CHK1). This approach, combining immunodepletion and add-back of recombinant forms of TOPBP1 with the DMAX assay, is documented in detail in a recent publication from our group (8). We prepared four samples, all contained TOPBP1-depleted XEE, and one sample contained unprogrammed IVTT lysate (blank), another had IVTT-produced full-length TOPBP1, and the remaining two had IVTT-produced Junior and III. We probed the blank and TOPBP1 samples with an antibody against TOPBP1, and as expected, we could see signal in the TOPBP1-containing sample, but not in the blank, and thus all detectable endogenous TOPBP1 had been removed from the XEE (Fig. 3*B*, left panel). DSBs were added and, after incubation, the samples were probed for P-CHK1. As seen in Figure 3*B*, right panel, both full-length TOPBP1 and Junior, but not III, could rescue the depleted XEE and activate ATR to produce P-CHK1. Thus, Junior, but not III, can activate ATR. But is this regulated ATR activation, which means Junior activating ATR only when DSBs are present? To answer this, we preformed assays containing Junior but lacking DSBs and found that in the absence of DSBs, Junior was unable to activate ATR (Fig. 3*C*). The data in Figures 2 and 3 make several important points. One, the region between BRCT2 and the AAD is dispensable for regulated ATR activation by TOPBP1. Two, BRCT7&8 is dispensable for recruitment of TOPBP1 to DSBs but critical for DSB-bound TOPBP1 to activate ATR. Three, the ability of TOPBP1 to bind DNA directly is dispensable for ATR activation, given that Junior activates ATR in XEE, but cannot bind DNA directly.

Our previous study had used misfolding mutants to examine roles for TOPBP1’s BRCT domains in ATR signaling at DSBs (8). In these experiments, we observed that misfolding of BRCT 7 or 8 caused attenuated DSB binding by TOPBP1, whereas we have shown here that loss of BRCT 7&8 has no impact on binding. Furthermore, our previous study showed that mutations in BRCT5 prevented ATR activation, and here, we have shown that the region spanning from BRCT 3 to 6 is dispensable for activation. Thus, in two cases, point mutations in BRCT domains behave differently than deletion mutants of the same domain. To explain this, we considered the possibility that point mutants may have a ripple effect, causing other regions of the protein to become nonfunctional. If so, then we expect that a deletion mutant for BRCT4&5 would be compliant for ATR activation, unlike our previous observations with point mutants (8). To test this, we removed BRCT domains 4 and 5 to create TOPBP1 ΔBRCT4&5 (Fig. 3*A*) and tested it for ATR activation. As shown in Figure 3*E*, TOPBP1 ΔBRCT4&5 can activate ATR. Thus, BRCT domains 4&5 are not formally required for activation, however, full-length TOPBP1-containing mutations in BRCT5 is compromised for ATR activation. Similarly, based on data shown in Figures 1 and 2, loss of BRCT7&8 does not impair recruitment, however, misfolding mutations in both BRCT 7 and 8 attenuate recruitment. It is thus clear that point mutations present with BRCT domains can impact the function of distal regions of the protein. The molecular basis for this ripple effect is currently under investigation.

### The sole function of BRCT0-2 region is to recruit TOPBP1 to DSBs

**Figure 4:**
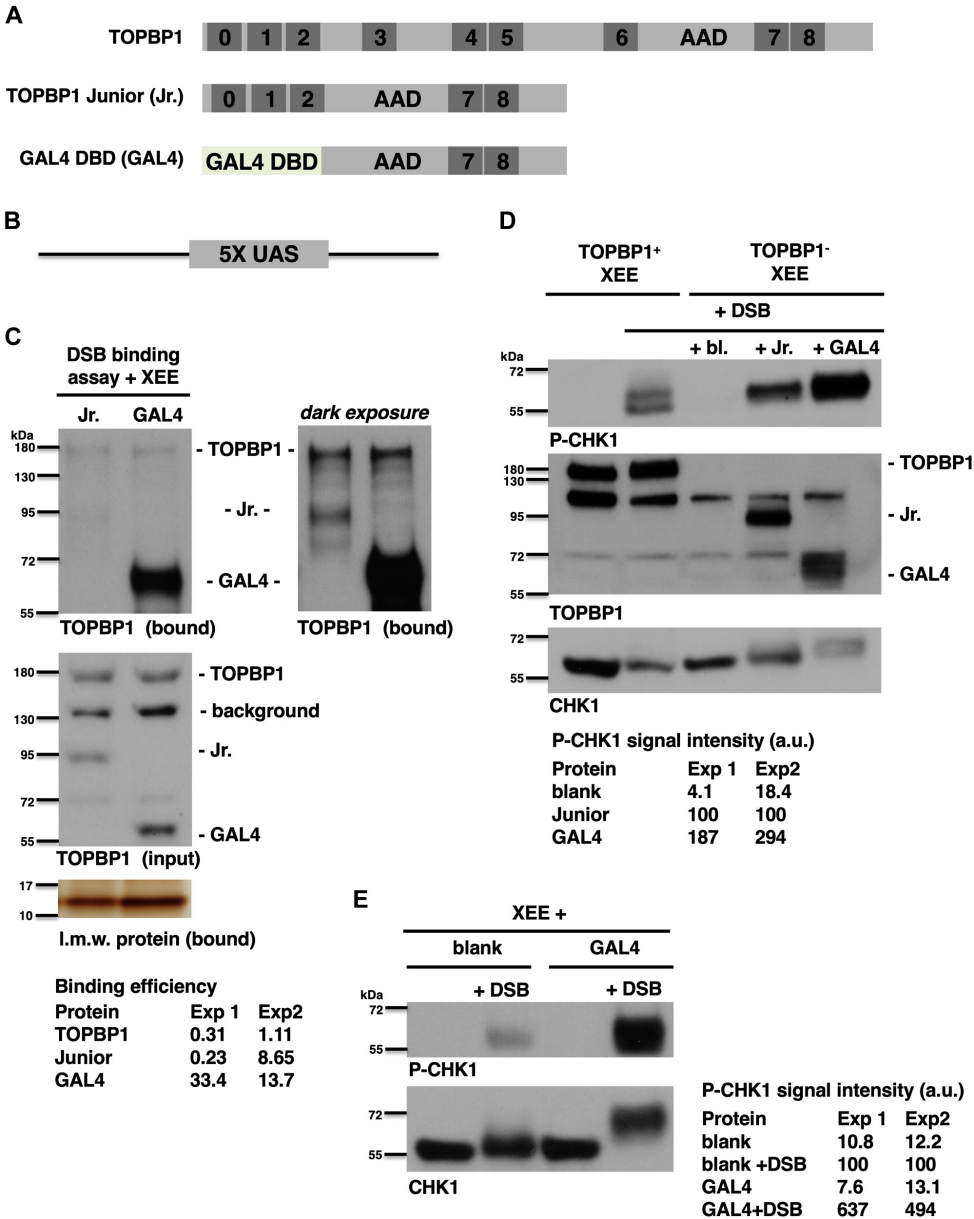
**Replacement of BRCT0-2 with a heterologous DNA-binding domain allows ATR activation.***A*, schematic showing the forms of TOPBP1 that were used for DSB-binding and DMAX assays. *B*, schematic showing the 5kb dsDNA “DSB” used for DSB-binding and DMAX assays. Five copies of the UAS are present, positioned in the center of the molecule. *C*, a representative experiment testing the ability of IVTT-produced TOPBP1 Junior, or the GAL4 derivative, to bind the 5XUAS-containing DSB is shown. The experiment was performed exactly as in Figure 1*C*. For the Western blot, we probed the samples with an antibody raised against the BRCT7&8 domains of *Xenopus* TOPBP1 (see (20)). This antibody, termed HU142, thus recognizes the two IVTT-produced proteins as well as the endogenous TOPBP1. We note that the blot labeled “input” shows that all three proteins of interest were present at similar levels in the total extract. We show two different exposures of the DSB-bound samples because of the intensity disparity between the GAL4 signal and TOPBP1/TOPBP1 Junior signals. The experiment shown is representative of two independently performed replicates. *D*, a representative experiment comparing the ability of TOPBP1 Junior and the GAL4 derivative to activate ATR is shown. The experiment was performed exactly as in Figure 3*B*, with the exception that we also included a sample of undepleted XEE, so that the efficiency of ATR activation by the two test proteins could be compared to that promoted by endogenous TOPBP1. The experiment shown is representative of two independently performed replicates. *E*, a representative experiment asking if the GAL4 derivative still requires DSBs to activate ATR. The experiment was performed exactly as in Figure 3*C*. The experiment shown is representative of two independently performed replicates. DSBs, DNA double-strand breaks; ATR, ataxia telangiectasia and Rad3-related; BRCT, BRCA1 C-terminal repeat; DMAX, DSB-mediated ATR activation in Xenopus; IVTT, *in vitro* transcription and translation; TOPBP1, Topoisomerase II binding protein 1; UAS, upstream activator sequence; XEE, Xenopus egg extract.

Our present study has defined a minimal TOPBP1 for ATR activation (BRCT0-2+ADD +BRCT7&8) and we have shown that BRCT0-2 plays a critical role in recruitment. We next asked if recruitment is the sole role for BRCT0-2 or if it performs an additional function, postrecruitment. To address this, we made another synthetic form of the protein, based on Junior, but we replaced BRCT0-2 with a heterologous DNA-binding domain, from the yeast GAL4 transcription factor (Fig. 4*A*). We also produced a 5kb linear dsDNA molecule containing five copies of the upstream activator sequence (UAS), the GAL4 DNA-binding site (Fig. 4*B*). Thus, with this design, we are targeting TOPBP1’s AAD and BRCT7&8 sequences to the DNA *via* the GAL4–UAS interaction. We first examined the ability of GAL4-ADD-BRCT7&8 to bind the UAS-containing DSB after incubation in XEE. A DSB-binding assay was performed with both IVTT-produced GAL4-ADD-BRCT7&8 or Junior, and we also probed for endogenous TOPBP1. As shown in Figure 4*C*, “input” panel, the amount of all three proteins present in the total extract was roughly equivalent. As seen in the DSB-bound fraction, however, the GAL4-ADD-BRCT7&8 protein was greatly enriched on the DSBs, relative to either endogenous TOPBP1 or Junior. Thus, GAL4-ADD-BRCT7&8 can efficiently bind to DSBs in this system. We next asked if GAL4-ADD-BRCT7&8 could activate ATR, using our DMAX assay. TOPBP1 was depleted from XEE, and the depleted extracts were supplemented with either blank IVTT or IVTT reactions programmed to produce Junior or GAL4-ADD-BRCT7&8. Figure 4*D* shows that ATR was efficiently activated by GAL4-ADD-BRCT7&8, and the P-CHK1 signal was stronger than that observed for Junior. The enhanced ability of the GAL4 derivative to activate ATR is consistent with the more efficient DSB-binding capacity of GAL4-ADD-BRCT7&8, relative to Junior (Fig. 4*C*). We also checked to see if GAL4-ADD-BRCT7&8 activation of ATR occurs in a regulated manner, such that it only happens when DNA damage is present. We found that DSBs are still required for ATR activation in extracts containing the GAL4 derivative (Fig. 4*E*). These data show that a heterologous DNA-binding domain can override the requirement for BRCT0-2 in ATR signaling, and thus that recruitment of TOPBP1 to DSBs is the sole function of the BRCT0-2 region.

### The role of BRCT7&8 is to multimerize the AAD

**Figure 5:**
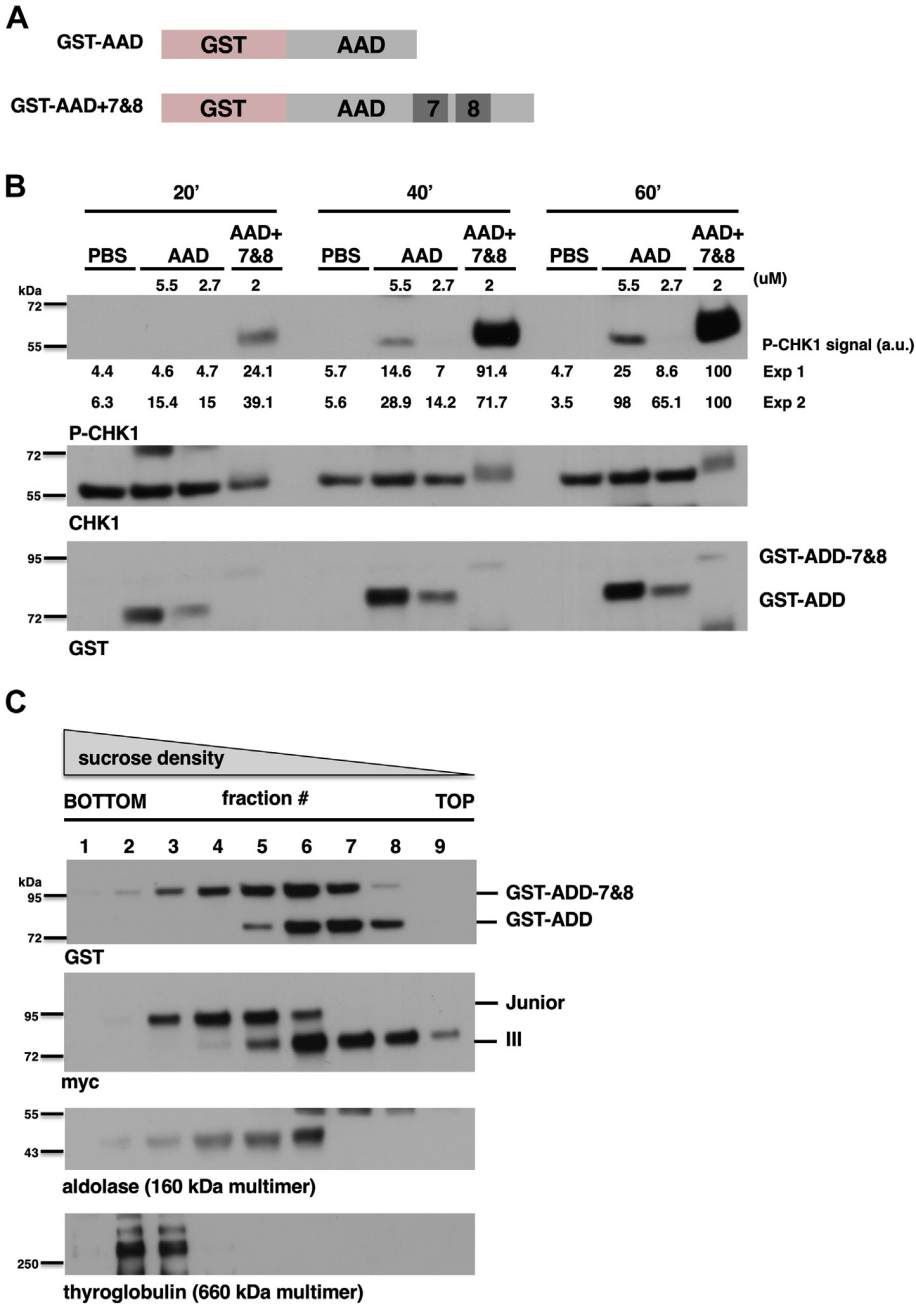
**Addition of BRCT7&8 to the AAD allows for more efficient activation of ATR.***A*, schematic showing the different GST fusion proteins that were used for ATR activation assays. *B*, a representative experiment testing the indicated GST fusions for ATR activation is shown. The indicated GST fusions were added to XEE at the indicated concentrations. Incubation was carried out for the indicated time and then the samples were processed for Western blotting and probed for the indicated proteins. The lanes-labeled “PBS” refer to samples that received PBS instead of a GST fusion protein. The experiment shown is representative of two independently performed replicates. *C*, Western blots of sucrose gradient fractions. Each gradient was divided into nine fractions, with fraction #1 representing the top and fraction #9 the bottom of the gradients. Proteins sediment within the gradient based on their molecular mass, with the higher molecular mass proteins sedimenting at the bottom. In the blots, we see that GST-AAD-BRCT7&8 sediments at a lower position than does GST-AAD, indicative of a higher molecular mass. We also see the same pattern when TOPBP1 Junior and III are compared. The experiment shown is representative of two independently performed replicates. AAD, ATR activation domain; ATR, ataxia telangiectasia and Rad3-related; BRCT, BRCA1 C-terminal repeat; TOPBP1, Topoisomerase II binding protein 1; XEE, Xenopus egg extract.

Data presented thus far have analyzed structural requirements for TOPBP1 during ATR activation. We have found that, besides the AAD, only the BRCT0-2 and 7&8 regions are needed for regulated activation to occur. Furthermore, BRCT0-2 can be replaced by a heterologous DNA-binding domain and ATR activation readily occurs. In addition, we have shown that the BRCT7&8 region is required for ATR activation, but not for recruitment to DSBs. These data suggest that, upon recruitment, the BRCT7&8 region acts together with the ADD to activate ATR. To test this possibility, we took advantage of previous data showing that addition of a GST-AAD fusion protein, at high concentration, to XEE results in DNA-independent activation of ATR (6). We reasoned that if BRCT7&8 acts with the AAD to stimulate ATR, then addition of the BRCT7&8 domains to GST-AAD would allow for more efficient activation (Fig. 5*A*). We, therefore, compared the effect of GST-ADD (at 2.7 μM and 5 μM) to that of GST-AAD-BRCT7&8 (at 2 μM) for ATR activation in XEE. As shown in Figure 5*B*, across all three timepoints, GST-AAD-BRCT7&8 was the better activator, despite its reduced concentration relative to GST-AAD alone. These data are consistent with experiments performed with purified human TOPBP1 derivatives, where an AAD-BRCT7&8 protein more efficiently activated ATR in reactions containing DNA compared to the AAD alone (26). We conclude that BRCT7&8 functions with the AAD to make activation of ATR more efficient. How might this be happening? One clue comes from recent work from our group (23), as well as from Cortez et al. (27). In these studies, it was shown that multimerization of the AAD is crucial for optimal ATR activation, and thus it may be that AAD-BRCT7&8 forms oligomers more readily than does the classic AAD. To explore this possibility, we examined how GST-AAD and GST-AAD-BRCT7&8 migrate in a sucrose density gradient, which gives a measure of a protein’s molecular mass and hence its multimeric state. Sucrose gradients were formed, ranging from 10%-40%, and purified GST proteins were loaded at the top. The gradients were then spun at a relative centrifugal force of 104,676*g* for 16 h. Fractions were then collected and probed for GST and two markers, thyroglobulin and aldolase. As shown in Figure 5*C*, GST-AAD migrated toward the top half of the gradient (fractions 5–8), whereas GST-AAD-BRCT7&8 sedimented deeper into the gradient (fractions 3–7), indicating that the molecular mass of GST-AAD-BRCT7&8 substantially exceeds that of GST-AAD. We note that monomeric GST-AAD-BRCT7&8 is just 25 kDa larger than monomeric GST-AAD, and this difference is unlikely to account for the difference in sedimentation observed within the sucrose gradients. We also compared myc-tagged TOPBP1 Junior to similarly tagged TOPBP1 III and observed the same pattern, whereby Junior sediments at a lower position than does III (Fig. 5*C*). Based on these data, we conclude that the addition of BRCT7&8 to the AAD allows for more efficient multimerization, and this explains why AAD-BRCT7&8 is a more robust activator of ATR than AAD alone.

## Discussion

### A minimal TOPBP1 for ATR signaling

**Figure 6:**
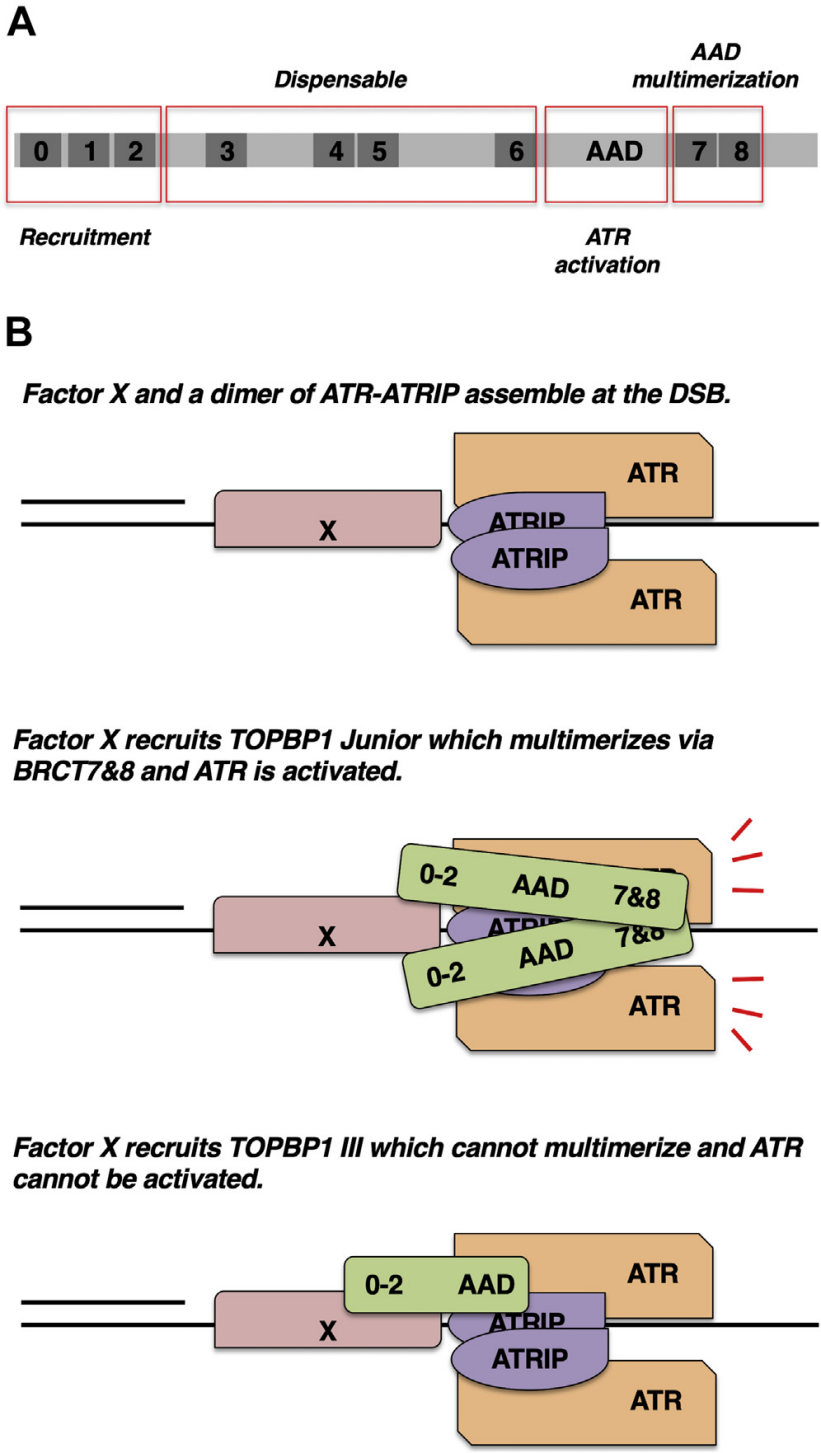
**Summary and a model for Junior-mediated activation of ATR.***A*, a schematic summarizing how the different regions of TOPBP1 contribute to ATR activation at DSBs. *B*, a model for why Junior, but not III, can activate ATR. Please see text for details. ATR, ataxia telangiectasia and Rad3-related; DSBs, DNA double-strand breaks; TOPBP1, Topoisomerase II binding protein 1.

TOPBP1 is a large protein with nine BRCT domains that has multiple functions within the cell. In this work, we have whittled down TOPBP1 to produce a minimal form that retains at least one major function—the ability to activate ATR, in a regulated manner, at DSBs. Surprisingly, a large amount of TOPBP1 sequence can be removed while still retaining the ability to activate ATR—at least 658 amino acids, or 43% of the protein are missing from Junior (Fig. 6*A*). In previous work from our group, we found that another reduced form of TOPBP1, termed Mini and comprised of the N-terminal half of the protein, is fully competent to initiate DNA replication (28). Later work went on to narrow the replication-promoting function of TOPBP1 down to the region spanning BRCT0-3 (29). Thus, for both of these critical functions (ATR signaling and DNA replication), TOPBP1 is a modular protein where discrete domains are devoted to a given function, and irrelevant domains can be removed without compromising function. In the case of DNA replication, all of the determinants are arranged in a linear manner at the N-terminus, whereas in the case of ATR signaling at DSBs, the determinants are spaced well apart from one another, on opposite ends of the molecule. We note that a previous study, using XEEs and a DNA substrate thought to mimic DSBs (70mers of polyA annealed to polyT and termed “AT70”), had suggested that the C-terminal half of TOPBP1 is sufficient for ATR activation (30). This would mean that the BRCT0-2 region is dispensable, which is difficult to imagine, however, it may be that the structural requirements for activation by the artificial AT70 substrate are different than those required for the much larger dsDNA molecules used here.

### The role of BRCT0-2

Our work shows that BRCT0-2 is important for TOPBP1 recruitment to DSBs (Fig. 1). Beyond BRCT0-2, there are other regions of TOPBP1 that can associate with DSBs, as the ΔBRCT0-2 (334–1513) mutant retains some binding activity (Fig. 1). However, our data show that BRCT0-2 is clearly sufficient for recruitment (Figs. 1 and 2). How is BRCT0-2 engaging with DSBs? Previous work had shown that this region of the human TOPBP1 protein can bind DNA directly (24) although others, using a different assay, did not detect DNA binding by BRCT0-2 (25). In our experiments, we observed that full-length TOPBP1 readily binds DNA in an assay containing IVTT lysate, but lacking XEE (Fig. 1*B*), whereas neither Junior nor III could do so. By contrast, when XEE is included in the assay, both Junior and III can now associate with DSBs (Fig. 1*C*). Our interpretation of these data is that BRCT0-2 controls recruitment of TOPBP1 to DSBs *via* protein–protein interactions with factors present in XEE and not *via* direct DNA binding. Having shown that BRCT0-2 is sufficient for recruitment, we went on to ask if this is the sole function of the region or if it plays an additional role, postrecruitment. We observed that replacing BRCT0-2 with a heterologous DNA-binding domain from the yeast GAL4 transcription factor allows ATR activation (Fig. 4), and thus the role of BRCT0-2 is limited to recruitment. An important research question now becomes what is the factor(s) that recruits TOPBP1 to DSBs? Over the years, numerous proteins that are known, or suspected, to be present at DSBs have been shown to interact with the BRCT0-2 region, and these include Treacle (31), NBS1 (32), CtIP (33), 53BP1 (34), MDC1 (35, 36, 37), RAD9 (38, 39), and RHINO (40). Thus, a major challenge for future work will be to identify the relevant protein(s) that recruit TOPBP1 to DSBs for ATR signaling.

### The role of BRCT 7&8

Our work highlights the crucial role of BRCT7&8 in ATR signaling at DSBs. We have shown that TOPBP1 Junior and III both bind to DSBs efficiently, but only Junior (which contains BRCT7&8) can activate ATR (Fig. 3). This suggests that BRCT7&8 functions postrecruitment to promote the AAD’s ability to activate ATR. This notion is reinforced by our finding that a GST-AAD-BRCT7&8 fusion protein activates ATR more efficiently than does GST-AAD alone (Fig. 5). These findings draw into question the very definition of the AAD. The AAD was originally defined by Dunphy et al., based on a series of GST-TOPBP1 fusion proteins that were used for *in vitro* kinase assays with ATR-ATRIP (6). One fusion, 972-1279 of the *Xenopus* protein, could stimulate ATR in this assay. In this same study, the authors went on to show that addition of a GST-TOPBP1 972-1279 fusion protein to XEE, at micromolar amounts, would activate ATR, even when no DNA was present in the system. This study thus defined the region of 972-1279 as a stand-alone AAD, and since then many other works have employed this same definition. More recently, Cortez et al. have narrowed this region down, again by combining GST fusions with *in vitro* kinase assays, and found that the region 1057-1173 of human TOPBP1 (1052–1167 in *Xenopus*) is a minimal AAD that activates ATR *in vitro* (41). These studies make it clear that the region originally defined as the AAD (“classic” AAD) can activate ATR, however, we have shown here that this region is not as efficient as a fragment that includes both the AAD and BRCT7&8 (Fig. 5). We have also shown that TOPBP1 Junior, which contains AAD-BRCT7&8, activates ATR in a regulated manner whereas TOPBP1 III, which contains just the classic AAD, does not activate ATR (Fig. 3). Thus, in two very different contexts, inclusion of BRCT7&8 with the classic AAD activates ATR more robustly than does the classic AAD alone.

What could BRCT7&8 be doing to make the classic AAD more robust? We and others have found that the AAD is better able to activate ATR when it assumes a multimeric form. Work from our group concluded that a tetramer is the optimal form of an oligomer (23), whereas others concluded it was a dimer (27). Regardless, it is clear that the monomeric form of the classic AAD has reduced activity. Thus, in this work, we considered the possibility that inclusion of BRCT7&8 to the classic AAD would allow for more efficient multimerization and that is indeed the case (Fig. 5). Taking these new data into context with previous data, we propose that the classic AAD will activate ATR when used at high concentration, as high concentration promotes multimerization, and that inclusion of BRCT7&8 sequences to the classic AAD lowers the concentration threshold for multimerization and thereby promotes more efficient activation of ATR relative to the classic AAD. We note that previous works have provided alternative possibilities for the role of BRCT7&8 in ATR signaling. For example, one previous study has implicated BRCT7&8 in ATR activation *via* its ability to interact with ATR that is auto-phosphorylated on T1989 (42). In this scenario, BRCT7&8 provides a contact to the ATR–ATRIP complex that would allow the classic AAD to stimulate ATR. Casting doubt on this scenario, however, is the fact that T1989 is not conserved in *Xenopus* TOPBP1, as well as other work showing that T1989 is not important for ATR signaling in mammalian cells (43). Yet another possibility is that BRCT7&8 functions together with a previously identified binding partner, the FANCJ/BACH1 protein (16). FANCJ is known to be present at DSBs and important for DNA end resection, and thus it may be that interaction between TOPBP1 and FANCJ produces a conformational change that allows the classic AAD to stimulate ATR. While this is certainly possible for events occurring on the DSB, it is unclear if this explains how BRCT7&8 helps the AAD activate ATR in the absence of DNA damage (Fig. 5).

### A model for TOPBP1 activation of ATR at DSBs

Our data have defined Junior as a minimal TOPBP1 for ATR signaling and suggest a model for how Junior activates ATR (Fig. 6*B*). This model is based in part on previous structural work showing that yeast ATR-ATRIP is a dimer of heterodimers (ATR-ATRIP::ATR-ATRIP, (44)). We propose that, upon chromosome breakage, an unknown factor, Factor X, and the ATR-ATRIP dimer assemble on the DSB (Fig. 6*B*). Factor X interacts with Junior’s BRCT0-2 region and recruits it to the DSB. Junior then uses its BRCT7&8 domains to drive interaction with an additional molecule(s) of Junior, and multimeric Junior then activates ATR. The early steps are similar for TOPBP1 III, however, III cannot multimerize efficiently and thus, despite being bound to the DSBs, monomeric III cannot efficiently activate ATR. How does this model explain the inability of TOPBP1 to activate ATR when there is no DNA damage present? We suggest that it is all about the amount of TOPBP1 that is present in the system. In XEE, the concentration of TOPBP1 is ∼37.5 nM and this is not high enough to allow soluble TOPBP1 to multimerize in a way that activates ATR. Upon induction of a DSB, however, TOPBP1 accumulates on DSBs, and hence its local concentration is increased, past a threshold required for multimerization. A similar pattern is observed for fragments of TOPBP1. The AAD alone has some propensity to multimerize and can activate ATR when present at very high concentrations (micromolar). Forced multimerization of the AAD greatly improves its ability to activate ATR (23, 27), and we have shown here that the BRCT7&8 domains play a key role in allowing the AAD to assume a multimeric form. Thus, in our model, the critical difference between soluble TOPBP1 and TOPBP1 that has accumulated at DSBs is the local concentration which, in turn, controls the multimeric state.

While our model for ATR activation at DSBs is consistent with available data, it is not yet clear if this represents a general mechanism for all forms of DNA lesions that activate ATR, such as stalled replication forks or ssDNA breaks, and further work is needed to resolve this important question. Further work is also needed to resolve the differential requirements for ATR activation observed for complex systems like XEEs and simple, fully purified systems such as those developed by Sancar et al (25, 26, 45). In the purified system, all that is needed for ATR-mediated phosphorylation of CHK1 is TOPBP1, ATR/ATRIP, and damaged DNA (26), or undamaged ssDNA plus RPA (45). In these studies, it was shown that a TOPBP1 fragment comprised of the AAD +BRCT7&8 is sufficient for ATR activation by damaged DNA (26), and thus the BRCT0-2 region is dispensable. On the other hand, in XEEs, the BRCT0-2 region is required for ATR activation (39). In the purified system, RPA-coated ssDNA is sufficient for TOPBP1 to activate ATR (45), however, in XEEs, 5′-DNA junctions on the RPA-ssDNA are also required and RPA-ssDNA alone is insufficient (13, 46, 47). It is likely that these differential requirements are due to the more complex environment of XEEs. In XEEs, multiple factors compete for access to DNA structures, and thus TOPBP1 requires BRCT0-2 and 5′-DNA junctions to gain access to the DNA, whereas in a purified system, there is no competition and thus these requirements are alleviated.

## Experimental procedures

### Materials

#### Plasmids

The following plasmids were used in this study. All constructs used in this study were derived from *Xenopus* TOPBP1 and the amino acid coordinates listed below are those of *Xenopus* TOPBP1. Details on construction are available upon request.

**Table:** 

Plasmid	Name	Vector	AA coordinates
myc-TOPBP1	Cut5	pCS2+MT	1-1513
myc-TOPBP1 334-1513	pHG33	pCS2+MT	334-1513
myc-TOPBP1 1-1279	pSY15	pCS2+MT	1-1279
myc-TOPBP1 BRCT0-2	pHG128	pCS2+MT	1-333
myc-TOPBP1 BRCT7&8	pKM1	pCS2+MT	1279-1470
myc-TOPBP1 Junior	pKM116	pCS2+MT	1-333 and 992-1513
myc-TOPBP1 III	pNB1	pCS2+MT	1-333 and 992-1279
myc-TOPBP1 ΔBRCT4&5	pMM113	pCS2+MT	1-479 and 759-1513
GAL4-AAD-BRCT7&8	pKR33	pCS2	GAL4 1-147 and TOPBP1 992-1513
GST-AAD E.coli expression vector	pHG117	pGEX-4T3	972-1279
GST-AAD-BRCT7&8 E.coli expression vector	pKR32	pGEX-4T3	972-1513

#### Recombinant proteins

The recombinant proteins used in this study were GST-AAD and GST-AAD-BRCT7&8. Both proteins were expressed in *Escherichia coli* BL21(DE3) cells at 37 °C for 4 h and purified from the soluble fraction according to standard procedures. Details can be provided upon request.

#### Antibodies

We used the following commercially sourced antibodies in this work: Myc (Millipore Sigma #M4439), GST (Millipore Sigma #05-782), CHK1 (Santa Cruz Biotechnology #sc-8408), P-CHK1 (Cell Signaling Technology #2341S), thyroglobulin (Santa Cruz Biotechnology #sc-53543), and aldolase (GeneTex #GTX101408). We also used our own antibody against *Xenopus* TOPBP1, HU142, which has been described (20).

### Methods

#### XEEs and immunodepletion

The high-speed supernatant (HSS) of XEE was used exclusively in this study. HSS was prepared exactly as described (48). For immunodepletion of TOPBP1, the HU142 antibody was used and the procedure was performed exactly as described (20). Depleted XEEs were then supplemented with IVTT-produced proteins (2.5 μl IVTT lysate in 20 ul of XEE) as described (8).

#### IVTT of recombinant proteins

IVTT reactions were performed using the SP6 TnT Quick Coupled Transcription/Translation System (Promega #L2080) according to the manufacturer’s instructions. Proteins were not purified after their production by IVTT, rather, the entire IVTT reaction was used as the source of a given protein.

#### DSB-binding assay with XEE

All DSB-binding assays containing XEE were performed exactly as described (8). To determine binding efficiencies, ImageJ software was used to quantify signal intensity for both bound and input signals that were exposed at the same time on the same blot, and the values were reflected as the ratio of bound to input.

#### DNA-binding assay with IVTT proteins

Twenty microliters of IVTT lysates were incubated with 5 μl of streptavidin beads linked to biotinylated 5kb PCR fragments. The DNA beads were prepared exactly as described (8) and 600 fmols of DNA were used per binding assay. After a 30-min incubation, the beads were collected on a magnetic stand and washed three times in PBS+0.1% TritionX-100. Bound proteins were then eluted with 2X SDS-PAGE sample buffer and examined by Western blotting.

#### DMAX assay

For DMAX assays, okadaic acid was first mixed with 20 μl of HSS to a final concentration of 1 μM, as described (8). Linear dsDNA derived from EcoRI-digested lambda DNA (8) was then added to the mixture and reactions were incubated at room temperature for 30 min. Samples were analyzed *via* Western blotting using standard conditions. For quantification of the P-CHK1 signal, ImageJ software was used to quantify signal intensity, the values for the control sample were adjusted to 100, and all other values were adjusted accordingly.

#### Sucrose density gradient centrifugation

Sucrose gradients (1.4 ml) were formed by layering 200 μl each of 10%, 15%, 20%, 25%, 30%, 35%, and 40% sucrose in egg lysis buffer salts (2.5 mM MgCl2, 50 mM KCl, 10 mM Hepes–KOH pH 7.7) and incubating for 2 h at room temperature and then 1 h at 4 °C. Samples were overlayed onto the gradients and centrifuged at 30,000 rpm for 16 h at 4 in a TLS55 rotor in a Beckman TL100 ultracentrifuge. Fractions were collected *via* bottom puncture of the tubes with a 21-guage needle. Molecular size standards were purified human thyroglobulin (GeneTex #GTX14718) and purified rabbit aldolase (Millipore Sigma A2714-500U).

## Data availability

All data are contained within the article.

## Conflicts of interest

The authors declare that they have no conflicts of interest with the contents of this article.
